# Evolution of dispersal and life history strategies – *Tetrahymena *ciliates

**DOI:** 10.1186/1471-2148-7-133

**Published:** 2007-08-06

**Authors:** Else J Fjerdingstad, Nicolas Schtickzelle, Pauline Manhes, Arnaud Gutierrez, Jean Clobert

**Affiliations:** 1 Laboratoire d'Ecologie, CNRS UMR 7625, Université Pierre et Marie Curie, Paris, France; 2 Now at the Department of Biology, Queens College, City University of New York, Flushing, NY, USA; 3 Biodiversity Research Centre, Université catholique de Louvain, Croix du Sud 4, 1348 Louvain-la-Neuve, Belgium; 4 Now at the Department of Biology, Indiana University, Bloomington, IN, USA; 5 Laboratoire d'Ecologie, CNRS UMR 7625, Université Pierre et Marie Curie, Paris, France; 6 Station d'Ecologie Expérimentale du CNRS à Moulis, Laboratoire Evolution et Diversité Biologique, Moulis, 09200 Saint-Girons, France

## Abstract

**Background:**

Considerable attention has focused on how selection on dispersal and other core life-history strategies (reproductive effort, survival ability, colonization capacity) may lead to so-called dispersal syndromes. Studies on genetic variation in these syndromes within species could importantly increase our understanding of their evolution, by revealing whether traits co-vary across genetic lineages in the manner predicted by theoretical models, and by stimulating further hypotheses for experimental testing. Yet such studies remain scarce. Here we studied the ciliated protist *Tetrahymena thermophila*, a particularly interesting organism due to cells being able to transform into morphs differing dramatically in swim-speed. We investigated dispersal, morphological responses, reproductive performance, and survival in ten different clonal strains. Then, we examined whether life history traits co-varied in the manner classically predicted for ruderal species, examined the investment of different strains into short- and putative long-distance dispersal, while considering also the likely impact of semi-sociality (cell aggregation, secretion of 'growth factors') on dispersal strategies.

**Results:**

Very significant among-strain differences were found with regard to dispersal rate, morphological commitment and plasticity, and almost all core life-history traits (e.g. survival, growth performance and strategy), with most of these traits being significantly intercorrelated. Some strains showed high short-distance dispersal rates, high colonization capacity, bigger cell size, elevated growth performance, and good survival abilities. These well performing strains, however, produced fewer fast-swimming dispersal morphs when subjected to environmental degradation than did philopatric strains performing poorly under normal conditions.

**Conclusion:**

Strong evidence was found for a genetic covariation between dispersal strategies and core life history traits in *T. thermophila*, with a fair fit of observed trait associations with classic colonizer models. However, the well performing strains with high colonization success and short-distance dispersal likely suffered under a long-distance dispersal disadvantage, due to producing fewer fast-swimming dispersal morphs than did philopatric strains. The smaller cell size at carrying capacity of the latter strains and their poor capacity to colonize as individual cells suggest that they may be adapted to greater levels of dependency on clone-mates (stronger sociality). In summary, differential exposure to selection on competitive and cooperative abilities, in conjunction with selective factors targeting specifically dispersal distance, likely contributed importantly to shaping *T. thermophila *dispersal and life history evolution.

## Background

Understanding the selective pressures affecting the evolution of dispersal strategies is of prime importance for a broad range of biological fields, ranging from conservation biology to research on the evolution of species, host-parasite interactions and communities of species [1-7]. Dispersal strategies of living organisms affect the dynamics, and the demographic and genetic structure of their populations [8,9], and impacts often crucially on the survival and reproductive success of individuals [2,10,11]. For example, dispersal has been theoretically demonstrated to profoundly influence the evolution of sociality [12-17] and dispersal strategies affect selection on other core life-history traits, e.g. reproductive strategies and effort, survival capacity, colonization ability, defenses against predators, parasites and diseases [7,18-23].

Co-variation among life-history traits has been the centre of much research in the past decades in the context of adaptive evolution as well as from the point of view of constraints on this evolution due to trade-offs between traits [24-29], and the existence of syndromes encapsulating dispersal strategies has been hypothesized [21,23,30,31]. For example, Baker and Stebbins [30] hypothesized that species living in unstable habitats in metapopulations with a high turn-over develop a set of co-adapted traits where high dispersal and colonization ability are linked to high fecundity and short life span (low survival), allowing excellent exploitation of freed-up patches (successful dispersal and colonization followed by rapid growth of the colonist population) [32]. Such hypotheses have been supported by correlative approaches, especially by comparing species in different types of habitats [23]. Theoretical models equally predict the evolution of syndromes in which dispersal ability influences or is influenced by the evolution of other life history traits [18,33], even if sometimes they come to a different conclusion than verbal or correlative approaches [34]. For example, Ronce and Olivieri [19] predicted a positive association between life span and dispersal while Crowley and McLetchie [35] predicted the reverse, the direction of the trend seemingly depending on details of the functioning of metapopulations [34] and also, we note, on potential constraints (trade-offs) on life-history traits and their capacity to evolve [25-28]. Similar complex theoretical results have also been found for the evolution of social traits and dispersal ability [17].

The evolution of dispersal syndromes through natural selection requires that the traits in question are under genetic determinism, and we must therefore demonstrate this to corroborate the theoretical view on dispersal syndrome evolution. Detailed studies on genetic variation in life history trait associations may also increase our understanding of the process of dispersal syndrome evolution, by allowing an evaluation of the degree to which the directionality of these patterns follow theoretical predictions or might be limited by trade-offs, and can also suggest additional selective factors that may have shaped syndromes. Nevertheless, only quite few studies test for and explore variation in dispersal syndromes apart from species comparisons, although genetic variance for dispersal itself has been demonstrated for some species (e.g. insects, birds, reptiles, mammals, [36-47]). Some correlative studies have found differences in the life-history of dispersing *versus *philopatric individuals [10,11] or intra-specific differences in dispersal strategies related with the cause of dispersal and landscape structure [48,49]. Other studies have found clear associations between individual propensity to disperse and some morphological, physiological or behavioral characteristics [50-54]. Such associations may, however, be due to maternal or environmental factors (i.e. forms of phenotypic plasticity, review by [27,47,55,56] as well as to genetic variation. Overall, if we except species with a clear dispersal-dedicated apparatus [24,45,47,58], evidence for genetic variation in dispersal syndromes and close dissections of trait associations are scarce (for a few exceptions [39,41,44], see also [38]). This means that our empirical and experimental insight into the evolution of these syndromes remains relatively poor.

One reason for the scarcity of evidence for genetic variation in dispersal strategies and other life history traits within species is that most studies have been done either on vertebrates, where assessing the genetic determinism of traits associations is difficult, or on invertebrates where an individual following up is often impossible. For this reason, artificial microcosms and clonal organisms with short generation time seem one of the best ways to investigate such syndromes ([23,59], see also [60]). Microorganisms, characterized by the general ease of maintaining large population sizes in the laboratory under controlled environmental conditions, should therefore be very well-suited organisms for studies on the co-variation between dispersal strategies and other core life history strategies. Surprisingly, however, very few studies have addressed the relationships between dispersal rate and core life history traits in microorganisms ([23], but see [60]).

We here present a study on the co-variation of dispersal strategies with other life-history traits in the unicellular, ciliated protozoan *Tetrahymena thermophila*. This small (60 μm) eukaryote feeds on bacteria and dissolved nutrients in fresh water ponds and streams in America [61,62], and while it is widely used as a model system by molecular and cell biologists [62], it has been surprisingly little studied by evolutionary biologists (but see [63-65]).

Yet the life history characteristics of *T. thermophila *make it particularly exciting for studies on dispersal. Firstly, this organism lives in habitats likely resembling those of meta-populations with high turn-over of local patches. Studies on life history trait associations in *T. thermophila *would therefore allow gaining insight into the degree to which central theoretical models can predict dispersal syndrome evolution in ruderal species (e.g. the classic colonizer syndrome model [23,30,31]). Secondly, genetic variation in all core life history traits can be easily estimated in *T. thermophila *because separate clonal lineages can be kept in the laboratory. Reproduction remains clonal whenever nutrients are present [66] and even under conditions inducing sexual reproduction (such as starvation), conjugation is impossible between clone mates because they carry the same mating type ([67], reviewed by [68]). Thirdly, *T. thermophila *shows an intriguing co-existence of apparent short- *versus *long-distance dispersal strategies (by normal cells versus cells that have transformed into elongated morphs with very numerous ciliae and a caudal flagellum [69]). *T. thermophila *of both morph-types swim about and explore their environment (personal observations), but the much greater swim speed (4–5 times [69,70]) and more directional movements (personal observations, see also [71]) reported for the elongated morph suggest that it is specialized for committed long-distance dispersal. Finally, *T. thermophila *cells form aggregations and secrete substances favoring the survival of other cells [72-74]; the evolution of dispersal strategies in this organism may therefore also be affected by a balance between kin-benefits and -competition (see also [16]).

Through experiments in the laboratory, we assessed variation in dispersal rate (in a two-patch system) and differences in the colonization capacity of single cells, and studied growth rate, patch carrying capacity, starvation resistance and concomitant changes in cell shape for ten strains of *T. thermophila*. These experiments and observations allowed us to (1) test for genetic variation and co-variation in core life history traits, (2) examine whether the directionality of life history trait associations across genetic lineages followed predictions for ruderal organisms (in particular as concerns the classic colonizer syndrome and competition-colonization co-existence) and (3) generate testable hypotheses on how the semi-social life style of *T. thermophila *may affect the evolution of its dispersal strategies.

## Results

### Growth from low density in presence of nutrients

All strains displayed a similar pattern of logistic growth, first exponential growth then reaching a plateau, where cells became smaller and rounder in a first time due to the rapid cell divisions and returned to their starting size and shape values once again when reaching the plateau. However, strains differed quantitatively. Growth rate and carrying capacity were largely strain-dependent (ΔAIC model with no effect versus model with a strain effect = 222.73), while variation between replicates within strain showed significant variation for some strains only and to a much lesser extent (ΔAIC model with a strain effect versus model with strain and replicate effects = 4.74). At the end of the experiment, where strains were at their carrying capacity, strains also differed in cell density (F_9,18 _= 10.72, P < 0.0001), size (F_9,18_= 17.47, P < 0.0001), and shape (F_9,18 _= 18.95, P < 0.0001).

**Figure 1:**
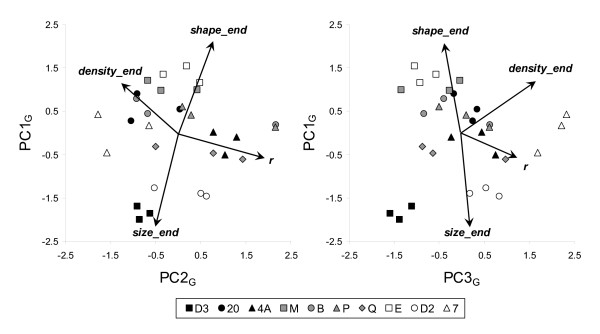
Principal component plots expressing correlations between cell morphology and growth variables in presence of nutrients for the ten *T. thermophila *strains studied. See text for details. PC1_G _expressed the opposition between strains with big round *versus *small elongated cells at carrying capacity. PC2_G _illustrated the *r versus K *strategy of growth, while PC3_G _represented overall growth performance.

Strains differed with respect to the co-variation among traits as well. This was shown by a principal component analysis on growth rate, final cell density, final cell size and final cell shape (carrying capacity *K *was dropped because it correlated tightly with final cell density), followed up by a discriminant analysis on the principal components (PCs). Such discriminant analyses test whether strains can be significantly distinguished from each other on the basis of the variables under study; they do not force artefactual strain separation [94]. All PCA components were significantly implicated in discriminating strains (PC1_G_: F_9,20 _= 48.55, P < 0.0001, R^2 ^= 0.96; PC2_G_: F_9,20 _= 2.45, P = 0.045, R^2 ^= 0.52; PC3_G_: F_9,20 _= 8.40, P < 0.0001, R^2 ^= 0.79) with 80 % of the replicates well classified within their strain. PC1_G _(explaining 46 % of the variance) represented a factor that was negatively associated with final cell size and positively with final cell shape (Figure 1). This means that at the carrying capacity some strains had small and elongated cells, others rounder and bigger cells. PC2_G _(explaining 27 % of the variance) represented a contrast between growth rate and final cell density (Figure 1). Thus, some strains grew rapidly in the beginning but reached only a low final cell density (*r *strategy), whereas others grew slowly in the beginning but reached a high final density (*K *strategy). PC3_G _(explaining 20 % of the variance) represented the overall performance of growth (*r *and *K*) from low density in the presence of nutrients, contrasting strains performing well (e.g. 7) to strains performing less well (e.g. D3) (Figure 1).

### Starvation in a medium devoid of nutrients

At the start of the experiment, cells were still dividing as if they were in a nutrient-rich environment. Although there was some variation between replicates and strains, a peak was observed at 8 h for density and cell elongation. Then density was steadily decreasing and cell shape rounding. Strains, however, differed significantly in survival rate, as estimated by survival as a density sum over time (F_9,20 _= 55.70, P < 0.0001). They also differed significantly as regarded cell elongation in response to starvation, as measured by mean (F_9,20 _= 90.96, P < 0.0001) and variance (F_9,20 _= 6.11, P = 0.0004) of maximal elongation, elongation persistence (F_9,20 _= 742.96, P < 0.0001) and frequency of disperser morphs (F_9,20 _= 15.80, P < 0.0001).

**Figure 2:**
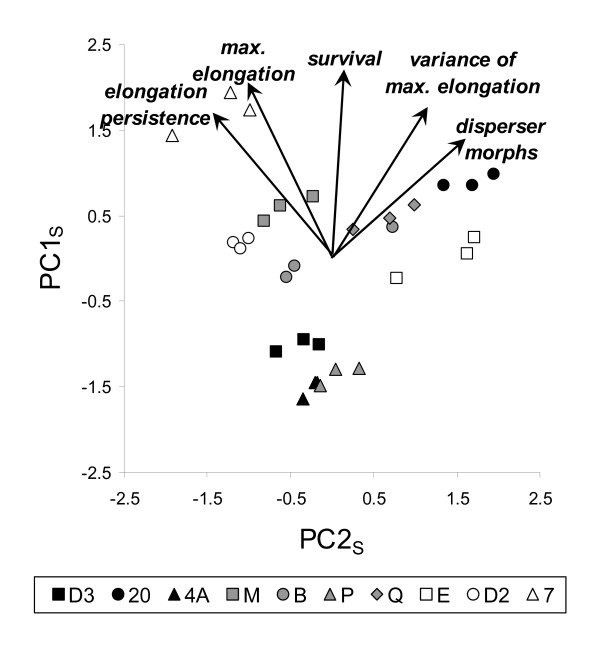
Principal component plot representing correlations between cell morphology and survival variables under starvation for the ten *T. thermophila *strains. PC1_S _is linked to overall survival and elongation capabilities. PC2_S _represented the cell elongation strategy, opposing strains where all cells elongates similarly for a long time to strains where some cells elongate more than others, up to becoming dispersal morphs.

Strains differed regarding the impact of starvation on trait associations, too, as was shown by a principal component analysis on cell survival, elongation, and production of disperser morphs, followed up by a discriminant analysis on the principal components (PCs). The first two PCA components explained 81 % of the variance in the data, were highly significantly implicated in discriminating strains (PC1_S_: F_9,20 _= 106.73, P < 0.0001; PC2_S_: F_9,20 _= 19.67, P < 0.0001) with all replicates well classified within their strain (P = 1). The first principal component, PC1_S_, (explaining 57 % of the variance) expressed the overall survival performance, cell elongation and production of disperser morphs in starvation conditions, positively associated with all five included variables (Figure 2). PC2_S _(explaining 24 % of the variance) represented a contrast of elongation reactions: strains with a larger and persisting mean maximal elongation but a limited variance in elongation (everyone elongates relatively similarly) versus strains with a larger variation between cells, some elongating far more than other, up to becoming real disperser morphs (Figure 2).

### Dispersal in presence of nutrients

**Figure 3:**
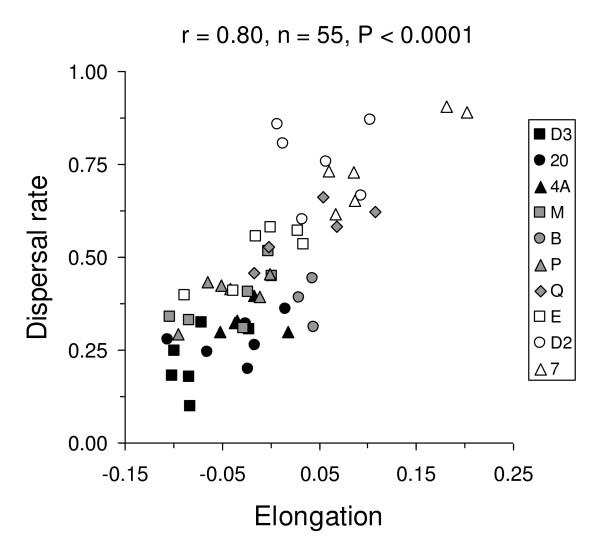
Correlation of dispersal rate and cell elongation in the dispersal experiment in presence of nutrients for the ten *T. thermophila *strains studied.

The rates at which *T. thermophila *cells dispersed from one tube (the start patch) through a connecting tubing to another tube (the target patch) were strongly positively correlated with the degree of cell elongation (Figure 3) and with the initial shape of cells(r = 0.34, n = 55, P = 0.011). A linear model analysis revealed that the differences in dispersal rate were primarily situated between strains (F_9,43 _= 10.64, P < 0.0001). When controlling for this strain effect, the correlation of dispersal rate with cell elongation was still significant (F_1,43 _= 21.46, P < 0.0001), but not the correlation with initial shape (F_1,43 _= 0.15, P = 0.701). Elongation also significantly differed between strains (F_9,43 _= 3.01, P = 0.007). Our tests hence showed that dispersal rates vary significantly among strains and also supported that elongation of *T. thermophila *cells is linked with dispersal.

### Single cell colonization capacity in presence of nutrients

Strains differed significantly as concerned the probability of successful colonization in new patches while replicates did not differ significantly (linear model with logit link and binomial error distribution: strain: X^2^_9 _= 71.42, P < 0.0001; replicate: X^2^_2 _= 0.000, P = 1.000; replicate*strain: X^2^_8 _= 5.93, P = 0.655; CV of strain means = 0.595).

### Correlation among life-history traits across experiments

**Figure 4:**
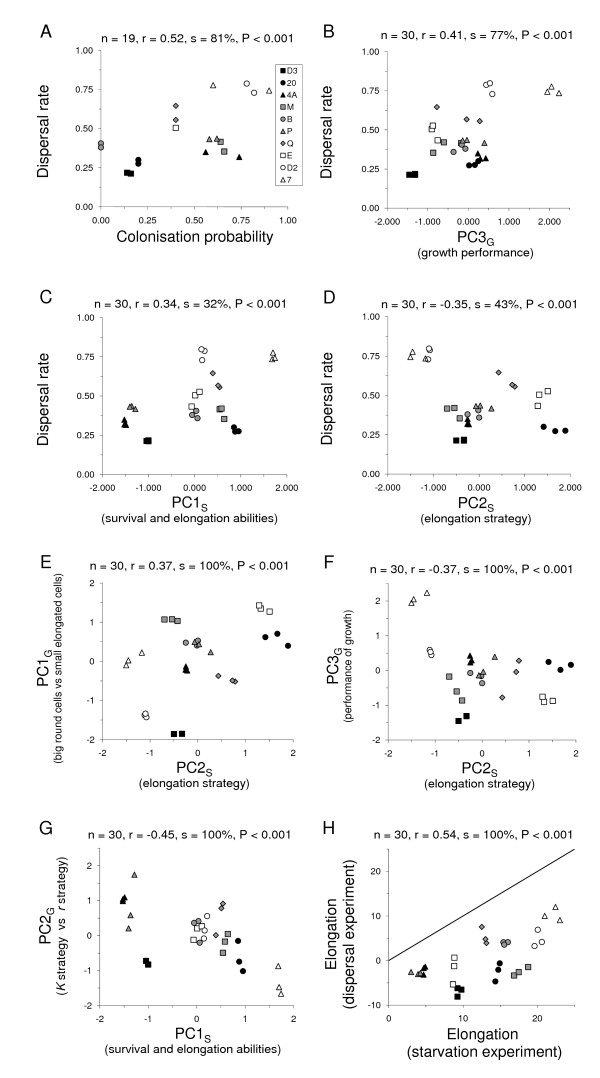
Correlation of life-history traits for the ten *T. thermophila *strains we studied. A) In the experiment on dispersal in presence of nutrients, a higher dispersal rate was associated with larger cell elongation. B) Strains with a high dispersal rate also had a greater probability of successfully colonizing a new patch as single cells. Strains adept at dispersing (C) and colonizing (D) grew faster and reached a higher final cell density in the presence of nutrients (PC3_G_). Elongation strategy (PC2_S_) was also associated to cell morphology and performance: strains producing more dispersal morphs and presenting a greater variance in elongation were characterized by small elongated cells (*versus *big round) at carrying capacity (E) and an overall inferior performance of growth in nutrient-rich medium (F). Strains presenting a *K *strategy of growth in nutrient-rich medium presented superior abilities to survive and elongate under starvation conditions (G). Note: because the variables whose correlation we studied do not come from the same experiment, each inset indicates the mean Spearman's correlation coefficient computed over 1000 random associations of replicates (r), the proportion of these associations where correlation was significant (s) and the probability to obtain this proportion by chance (P). See text for detail. Graphs, however, display means of five random associations to illustrate within strain variation.

Across experiments, strains that showed a great dispersal rate in the two-patch dispersal experiment in presence of nutrients also had a greater probability of successfully colonizing new patches as single cells (Figure 4A). Dispersal and colonization capacities were not associated with the cell sizes and shapes attained at carrying capacity after growth from low density in the presence of nutrients, as shown by their non-significant co-variation with PC1_G _(dispersal rate: mean Spearman correlation coefficient r = -0.04, n = 30, proportion of significant correlations s = 0%, P = 1; colonization probability: r = -0.06, n = 19, s = 0%, P = 1), nor with the trade-off between growth rate and cell density at carrying capacity (PC2_G_) (dispersal rate: r = -0.09, n = 30, s = 0%, P = 1; colonization probability: r = 0.20, n = 19, s = 3%, P = 0.998). However, dispersal rate and colonization probability were positively associated with the overall growth performance (PC3_G_) (dispersal rate: Figure 4B; colonization probability: r = 0.46, n = 19, s = 50%, P < 0.001). Dispersal rate was positively associated with the overall survival capacity and trend to elongate when faced with starvation in a nutrient-free medium (PC1_S_: Figure 4C), but colonization probability was not (r = 0.03, n = 19, s = 0%, P = 1). Dispersal rate and colonization probability in a nutrient-rich medium were higher for strains where all cells elongated similarly and for a long time but did not produce dispersal morphs, as shown by a negative association with PC2_S _(dispersal rate: Figure 4D; colonization probability: r = -0.51, n = 19, s = 76% P < 0.001). This elongation strategy (PC2_S_) was also associated with strains presenting big round (versus small elongated) cells at carrying capacity (PC1_G_: Figure 4E) and to the overall performance of growth in nutrient-rich medium (PC3_G_: Figure 4F). Furthermore, strains showing high carrying capacities but low growth rates (*K *strategy strains) in presence of nutrients also showed superior abilities to survive and elongate under starvation conditions (Figure 4G). Finally, elongation in nutrient-rich and nutrient-free (starvation) conditions were highly correlated (Figure 4H) but elongation was always greatly more pronounced under starvation conditions.

**Figure 5:**
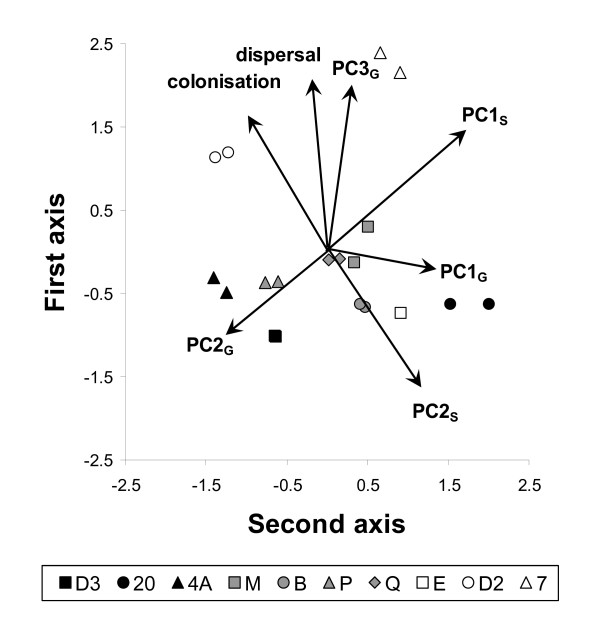
Summary principal component plot representing the correlations between the seven variables summarizing the four experiments. Graph displays means of five random associations to illustrate within strain variation; see text for detail.

The above associations were well represented by the first two axes of a final principal component analysis (Figure 5) performed on the seven variables summarizing the four experiments (dispersal rate, colonization probability, survival and elongation in presence of nutrients PC1_G_, contrast between growth rate and final cell density PC2_G_, growth performance PC3_G_, survival and elongation under starvation PC1_S_, elongation strategy PC2_S_; Figure 4). The first axis of this comprehensive PCA explained on average 39% of the variance (SD: 1.6%; range: 34% to 44%, over 1000 random associations of replicates across experiments) and the second axis 23% of the variance (SD: 1.4%; range: 19% to 28%). These two axes allowed discriminating strains very efficiently, with only on average 16% (SD: 7.7%; minimum, median, maximum: 0%, 16%, 37%, respectively) of the replicates not correctly classified. Some strains (e.g. 7 and D2) were always perfectly discriminated, and misclassifications only concerned a single replicate for the majority of the strains; only B strain was more frequently misclassified for all replicates.

## Discussion

### Genetic variation in dispersal and life history strategies

Our study provided strong evidence for genetic variation in dispersal and life history strategies in *Tetrahymena thermophila *protozoans. Strains varied considerably and significantly in nearly all aspects of life history (growth performance, survival, cell morphology changes, and degree of phenotypic plasticity of cell shape) under different environmental conditions, and showed significant differences in the associations of these traits under our experimental conditions (axenic food resource or food free medium). Life history trait differences were in many cases significantly related with dispersal strategies, which also varied significantly between strains. Among-strain differences in life-history traits and trait associations were not just due to variation in strain quality, although some *T. thermophila *strains did perform better than others as regarded a series of core fitness traits (survival, dispersal, growth performance, and colonization ability). This is because strains also differed with respect to various trait associations that cannot as such be considered to represent differential quality but rather different strategies. Examples are the observed among-strain differences in growth rates *r versus *final population density in presence of nutrients (PC2_G_), cell shape *versus *size in presence of nutrients (PC1_G_), and the opposition between a high degree of phenotypic plasticity of cell shape (high variance in cell elongation) with some cells turning into dispersal morphs *versus *a more durable and greater, but less plastic, mean cell elongation under starvation (PC2_S_). The *T. thermophila *ten strains hence differed also with respect to the patterns of their investment along different trait axes. Hence, our study adds to the limited evidence (*e.g*. [24,38,39,41-47,58]) for genetic variance in dispersal syndromes within a species. Also, the among-strain variance in the degree of plasticity of cell shape found in *T. thermophila *is similar to recent findings of genetic variance in plasticity for core life-history traits in other animals, e.g. *Caenorhabditis elegans *nematodes [55] and in dispersal related morphological traits in pea aphids (a winged-non-winged polyphenism) [47].

The life-history trait association differences found among *T. thermophila *strains should be attributed to among-strain differences in genes of the macronucleus (somatic nucleus characteristic for ciliated protozoans; reviews by [75,76]), not directly the micronucleus (germline nucleus, not transcribed [77]). This is because macronucleus genes represent a modified, often variably amplified and rearranged subset of micronucleus genes [75,76,78]. The genetic trait variance expressed among clonal lines is therefore not necessarily the same that would be found in crossing experiments (sexual reproduction; involving micronuclei). Another curiosity of ciliates, the amitotic divisions of macronuclei making daughter cells from a clonal division potentially have slightly different alleles or amounts of alleles ([78], reviews by [75,76]) did not invalidate our study. Such differences among cells within a clone line would only make our tests for genetic differences among strains more conservative. Equally, non-genetic 'maternal' effects are not likely to have contributed importantly to among-strain differences and so confounded our study, because all strains were maintained under the same conditions for some 700 generations before the start of experiments (> one year). Finally, the observed strain differences in morphological responses to starvation were very unlikely to be artefactual. We used standard techniques (centrifugation, decanting of super-natant and addition of water) to eliminate nutrients from the medium [e.g. [62,69]], and any damage during centrifugation would rather lead to ciliae loss and so to a decreased, not a greatly enhanced swimming speed. Cells, moreover, started swimming within seconds after centrifugations indicating perfectly preserved cell integrity, verified also under the microscope (E.J.F and N.S. personal observations). Also, all strains were subjected to precisely the same manipulations (same centrifugal forces and length of centrifugation).

### Covariation of dispersal strategies with life history traits

#### Dispersal strategies, growth performance, and survival

The association of *T. thermophila *dispersal rates in presence of nutrients with other life history traits fits partly with the classic 'colonizer syndrome' [30] envisioned typical for species living in variable patches in metapopulations (see also [21,23,31]). Strains with great short-distance dispersal rates in presence of nutrients had a better growth performance, and high colonization abilities as predicted ([30,31,79] but see [80]), but contrary to classical predictions they also had a better mean strain survival rate than did less dispersive strains. An actual negative co-variance (trade-off) between two life-history traits could be masked by differences among strains in overall condition, because some strains would have more resources to invest both in growth and in survival [28,29]. Our experiments were not set up to explicitly explore this issue. All our strains were, however, kept in the same conditions of nutrient availability in the growth experiments, and so should have had the same amount of resources available. Also we note that our findings could be explained by some alternative dispersal evolution models that do predict positive associations of survival with dispersal rates and reproductive effort for ruderal species, the precise expectations depending on landscape characteristics (productivity, demographics, spatio-temporal structure [34], review by [23]), that are only little known for *T. thermophila*.

The opposite end of the spectrum, namely *T. thermophila *strains dispersing little, growing less, and colonizing less well as single cells in presence of food, and surviving food-stress poorly, represented relative philopatry as long as the environment remained good, and likely long-distance dispersal (via fast-swimming dispersal morphs) when the environment turned bad. Such a long-distance colonizer strategy could allow coexisting with better competitor strains (see above). Alternatively, 'philopatric' strains may come from habitats with less local spatio-temporal variability (see e.g. [23,32]) but more catastrophic patch degradations (favouring the production of the dispersal morph). Finally, the variation among strains in dispersal strategies and life history could represent non-adaptive variance around an adaptive mean. In all cases, relative philopatry in presence of food did not signify an adaptation to a more intensive exploitation of already colonized patches (see e.g. [81,83,84,86]), because the growth performance of philopatric strains was inferior and also philopatry was not related significantly with *r *versus *K *strategies. Nevertheless, the lower dispersal rate of philopatric strains suggests that they freely tolerate higher densities without dispersing massively. Density-dependent dispersal strategies are indeed predicted by several theoretical models and have been reported in a number of species (e.g. [4,85-88]).

Potential density-dependence of dispersal strategies is particularly interesting given the primitive form of cooperation exhibited by *T. thermophila*. Cells secrete certain chemical compounds ('growth substances') that increase the survival of other cells [72,73] and also actively form aggregations [74], which should enhance the effective concentration of these substances. Those *T. thermophila *strains that disperse little even when at high density and are less good at colonizing as single cells, could be strains adapted to and dependent on greater cooperation than the more dispersive strains. Consistent with this, strains producing many dispersal morphs (generally philopatric strains) have smaller cell sizes (at carrying capacity; Figure 4E), and so each cell likely has fewer resources than is the case in more dispersive strains. The inferior rates of survival under starvation for philopatric strains corroborate this idea. If our hypothesis is correct, the philopatric strains should show a stronger degree of aggregation and only disperse much when densities are very high and benefits of 'cooperation' outweighed by costs of conspecific competition. We are currently testing this.

#### Dispersal morphs

Morphological differences between dispersers and non-dispersers were found in our study, as in a series of other species ([41,58,79], see also [10,11]). Morph diversity was, however, greater than suggested by previous morphological studies on *T. thermophila *[69]. Dispersing cells were more elongated than non-dispersers in our two-patch dispersal experiment, as expected, and strains that elongated much in presence of nutrients also showed greater mean cell elongation under starvation (but a lower variance and hence lower plasticity). Elongated disperser cells, however, differed in shape from the more elongated cells produced under starvation, and strains dispersing much in presence of nutrients surprisingly produced only few of the fast-swimming dispersal morphs described by Nelsen and Debault [69]. Importantly, the differential production of dispersive morph types by different strains was linked to variation in core life history trait associations (growth, survival, colonization, short-distance dispersal), with strains performing poorly under normal conditions producing more fast-swimming dispersal morphs under starvation.

That the fast-swimming very elongated dispersal morphs likely constitute a commitment to long-distance dispersal in degraded environments was supported by our study. Firstly, our dispersal experiment showed that normal cell morphs or partially elongated cells were very capable of short-distance dispersal [69,70]. Secondly, our starvation experiment confirmed that considerable time is required for a cell to transform itself into the very elongated dispersal morph (4–8 hours in this study, which corresponds to half a generation; see temperature dependent data in [69,70]). Hence transformation, likely also energetically costly, should only occur when cells need to surpass the mobility capacities of normal or partially elongated morphs. Strains producing many fast-swimming morphs should therefore benefit from a dispersal-distance advantage, which may help explain the coexistence of low performance strains with strains doing well (see-colonization-competition models for coexistence [81], see also [82] and references in these).

Other selective factors could also have contributed to the observed variation among strains in dispersal distance strategies. For example different strains may come from habitats that vary with respect to the selective pressures of kin competition *versus *spatio-temporal variation in habitat quality, distributions of resources and kin in space, or the shapes of distance-dependent dispersal cost functions (e.g. [17,21,81,82,86,87,89]; see also [85]). Finally, we cannot reject that strain variation in life history traits could represent non-adaptive variation around an adaptive mean strategy. Intimate knowledge on the habitats of origin of different strains or, better still, experimental evolution studies will be required to test this.

## Conclusion

We found strong evidence for genetic variation in dispersal syndromes in *T. thermophila *protozoans, because strains differed in overall life-history associations, with dispersal strategies, colonization capacity, survival, reproductive performance, and cell shape plasticity showing complex patterns of variation. While some strains fit rather well with the classic colonizer syndrome (high short-distance dispersal rates, high colonization capacity, and elevated growth performance), they were also characterized by good survival abilities and produced few of the putative long-distance dispersal morphs when subjected to environmental degradation. Poorly performing, locally philopatric strains, by contrast, produced relatively many of these fast-swimming dispersal morphs, and so likely benefit from a dispersal-distance advantage which may facilitate their persistence. Finally, the smaller cell size of these latter strains at carrying capacity and their poorer skills at colonizing as individual cells, suggest that they may be adapted to greater levels of dependency on clone-mate cells (stronger sociality). Overall, differential exposure to selection on competitive and cooperative abilities, in conjunction with selective factors targeting specifically dispersal distance, likely contributed importantly to shaping *T. thermophila *dispersal and life history evolution. Ongoing studies on aggregation behavior and density-dependence of dispersal strategies will explore this further.

## Methods

### Culture conditions, and strain conditioning

Stocks of 10 *T. thermophila *strains (Table 1) were maintained in an axenic (bacteria-free) culture medium (2% proteose peptone, Difco no. 3; 0.2 % yeast Difco; demineralized water; all autoclaved after mixing) (see also [62]), and new cultures were made every three to five weeks by transferring 500 μl of an old stock to a 50 ml tube filled with 40 ml of new culture medium. All work was done under sterile conditions under a flow-hood. Culture tubes were kept at 27°C at a 12 h light/12 h dark cycle in an incubator with sun-glow bulbs emitting light corresponding precisely to natural sunlight.

**Table 1:** *Tetrahymena thermophila *strains used

Name	Distributor and reference	Geographic origin	Isolator and isolation date
D2	F. P. Doerder 18282-4	PA, USA – pond CRWP	F. P. Doerder, 2002
D3	F. P. Doerder 18296-1	PA, USA – pond SG29	F. P. Doerder, 2003
4A	CCAP 1630/4A	Unknown	Unknown
M	CCAP 1630/M	Unknown, USA	A. Phelps, 1948
P	CCAP 1630/P	Unknown, USA	A. Phelps, 1948
Q	CCAP 1630/Q	Unknown, USA	A. Phelps, 1948
20	CCAP 1630/20	Unknown	Unknown
B	ATCC 30384 (B-18687)	Woodshole, MA, USA	E.M. Simon & D.L. Nanney, 1952 S.L. Allen
E	ATCC 205043 (ME 44w)	Maine, USA – McCurdy Pond, Pemaquid.	E.M. Simon & D.L. Nanney, 1986
7	ATCC 30306 (1726 A1)	Woodshole, MA, USA	D.L. Nanney, 1953.

CCAP [90]; ATCC [91]; P. Doerder: strains collected by F. P. Doerder, Cleveland State University, Ohio, USA. All CCAP strains mated with at least one ATCC or Doerder strain, proving that they were *T. thermophila*.

### Data collection using digital imaging

**Figure 6:**
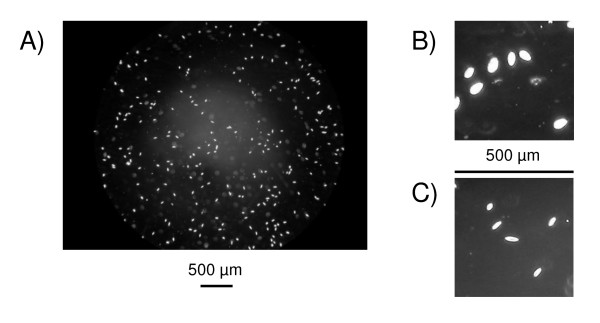
A) Typical digital picture of *Tetrahymena thermophila *cells used to extract quantitative variables. B) Cells (D2 strain) in normal growth condition (with nutrients) showing large size and a largely rounded shape. C) Cells (20 strain) eight hours after nutrient removal, showing reduced size and (middle cell) the typical elongated shape of the fast-swimming dispersal morph described by Nelsen and Debault [69].

A procedure based on image analysis was developed to collect data from digital pictures taken under the microscope. For each sample to be measured, aliquots of 10 μl were taken by pipette after vortexing for 10 seconds at medium speed to homogenize the cell suspension. Each aliquot was then placed in a chamber of a Plexiglas cell count slide (KOVA Glasstic Slide 10 no grids from Hycor Biomedical Inc., California, USA, reference 87146). One grey level picture was taken per chamber using a digital camera (Nikon Coolpix 4500; settings: focal length at 8 mm, fixed exposure at 1/60 s, f/6, manual focus fixed to infinity with focus obtained manually using the microscope) mounted via an eyepiece (Olympus Coolpix digital coupler) on a microscope (Olympus CX-41; settings: 10 × oculars, 4x/013PhL planar lens, condensator placed on phase 2, maximum light); an example of such a picture is shown in Figure 6. Conversion of measurement units on pictures (pixels) into real units (μm and μm^2^) were obtained from a picture made with the same setup of a similar cell count slide with a calibrated grid of known size and volume (KOVA Glasstic Slide 10 with counting grids, reference 87144).

Pictures were analyzed using the public domain image-analysis program Scion Image [92] with a macro that we developed to automate the treatment of large numbers of pictures. The macro first converted each grey level pixel into black or white, with the threshold set at 200 as it proved to optimize discrimination of cells from dust and scratches. Then the "Analyze Particles" function was used to export to a text file a list of parameters for each particle present on the picture. Particles smaller than 35 or bigger than 500 pixels were discarded because these sizes are far outside normal sizes for *T. thermophila *cells [62] and hence represented dust. For each cell, the following variables were measured: size, color density (mean, mode and std; using the grey level picture), position (X and Y coordinates), perimeter, and three variables from the best fitting ellipse: major axis, minor axis and angle (angle between the major axis and a line parallel to the x-axis of the image). A shape variable was computed as the ratio major/minor axis for each cell; a perfect circle had shape = 1 and shape increased for more elongated cells. The density of cells per picture was obtained from the number of particles identified.

To ensure that results were comparable between all the pictures, the method was designed using fixed parameters only, both for taking the pictures (microscope and camera parameters) and analyzing them (Scion Image macro parameters) with settings described above. Extensive fine-tuning of each step was performed to define an optimal method, and results of picture analyses were manually checked on several hundreds of cells to verify the validity of measures recorded. Artefacts (missed cells, or dust considered as cells) were less than 5% of the results and distributed randomly among pictures; therefore they did not bias the results even if they did increase the random noise in the data.

### Experimental design

For each of our 10 *T. thermophila *strains (Table 1), we studied (1) growth from low density in presence of nutrients, (2) survival in a medium devoid of nutrients (starvation), (3) dispersal in presence of nutrients, and (4) single cell colonization capacity in presence of nutrients. Experiments were started from cell cultures inoculated one week before. 10 pictures of the initial tube were taken and density of cells was measured so that experiments could be started with a precise cell density by diluting the solution accordingly. All experimental setups were autoclaved before experiments and conserved in the incubator under the same conditions as strain stocks (see above).

### Growth from low density in presence of nutrients

Growth rate and carrying capacity in nutrient-rich conditions were examined by placing approximately 50 000 cells in a 12 ml polypropylene culture tube (Greiner Bio-One), adding nutrient medium till a total volume of 5 ml to reach a starting cell density of 10 000 cells/ml. Three replicate tubes were made per strain. Pictures were taken at six different times (from 0 to 192 h).

From the raw cell measurements at the replicate level, we estimated five major variables describing cell population growth: carrying capacity *K *and growth rate *r*, and final (i.e. at carrying capacity) cell density, size and shape (Table 2). Growth was estimated by fitting the following logistic growth function to successive densities *D*_*t *_over time *t *(*t *> 0):

**Table 2:** List of variables used in statistical analyses

Experiment	Variable name	Definition	Estimation method and level
Growth	K	Carrying capacity (see eqn 1)	Logistic function fitted at the replicate level to successive densities (time > 0) estimated at the picture level (5 values per time step)
	r	Growth rate (see eqn 1)	Logistic function fitted at the replicate level to successive densities (time > 0) estimated at the picture level (5 values per time step)
	Final cell density (at K)	Mean density of cells at the last time step (192 hours)	Mean of 5 pictures per replicate
	Final cell size (at K)	Mean size of cells at the last time step (192 hours)	Mean of all cells of the 5 pictures per replicate
	Final cell shape (at K)	Mean shape of cells at the last time step (192 hours)	Mean of all cells of the 5 pictures per replicate
Starvation	Survival as a density sum over time	Area between the density curve and the density at time = 0 line, starting at time = 8 h which was the mean peak density for all strains	Curves defined by successive values through time of the mean of the 5 pictures per replicate
	Mean maximal cell elongation	Difference between longest shape and start shape, expressed in percentages of start shape	Estimated on means of all cells in the 5 pictures per replicate
	Variance in maximal cell elongation	Difference between the 95th upper percentile and the mean of the shape distribution at the time when the maximal elongation is reached, expressed as percentage of the same difference at start	Estimated on means of all cells in the 5 pictures per replicate
	Elongation persistence	Sum of elongation over time, i.e. area between the shape curve and the shape at time = 0 line, starting at time = 8 h	Curves defined by successive values through time of the mean of the 5 pictures per replicate
	Frequency of putative disperser morphs	Frequency of disperser morphs observed at time = 8 h	Mean of the 5 values per replicate (recorded on each sample just after picture was taken)
Dispersal	Initial cell shape	Mean shape of cells in the initial tube	Mean of all cells of the 10 pictures per block
	Dispersal rate	Proportion of cells in the target tube at the end of the experiment	Computed as a combination of parameter values at the replicate level
	Cell elongation	Difference between mean cell shape in target tube and start tube, expressed as percentage of the shape in start tube ^1^	Computed as a combination of parameter values at the replicate level
Colonization	Colonization probability	Proportion of tubes where a cell population developed from the single cell added to the tube	

^1 ^Estimating cell elongation relative to initial shape gave nearly identical results (results not shown).

Dt=D0⋅KD0+(K−D0)⋅e−r⋅t
 MathType@MTEF@5@5@+=feaafiart1ev1aqatCvAUfKttLearuWrP9MDH5MBPbIqV92AaeXatLxBI9gBaebbnrfifHhDYfgasaacH8akY=wiFfYdH8Gipec8Eeeu0xXdbba9frFj0=OqFfea0dXdd9vqai=hGuQ8kuc9pgc9s8qqaq=dirpe0xb9q8qiLsFr0=vr0=vr0dc8meaabaqaciaacaGaaeqabaqabeGadaaakeaacqWGebardaWgaaWcbaGaemiDaqhabeaakiabg2da9maalaaabaGaemiraq0aaSbaaSqaaiabicdaWaqabaGccqGHflY1cqWGlbWsaeaacqWGebardaWgaaWcbaGaeGimaadabeaakiabgUcaRmaabmaabaGaem4saSKaeyOeI0Iaemiraq0aaSbaaSqaaiabicdaWaqabaaakiaawIcacaGLPaaacqGHflY1cqWGLbqzdaahaaWcbeqaaiabgkHiTiabdkhaYjabgwSixlabdsha0baaaaaaaa@48D9@

with *D*_0 _being the initial density at time 0, and two parameters to be estimated: growth rate *r *and carrying capacity *K*. The fitting was achieved through least-squares non linear programming using PROC NLP in SAS [93]. Statistical difference between replicates and strains were tested by comparing a model with equality constraints of *K *and *r *parameters between replicates of each strain, a model with equality constrains of *K *and *r *parameters between strains, and a model where parameters *K *and *r *were free to vary between strains and replicates; model selection was achieved using the AIC criterion [94-96].

### Survival in a medium devoid of nutrients (starvation)

To remove all traces of nutrients from cell cultures, the contents of five tubes per strain were concentrated and washed with water (similar to [70]). This was done by centrifugating tubes at 2000 r.p.m. for three minutes, after which the supernatant (approximately 35 ml) was aspirated using a vacuum pump. The pellet, containing the cells, was then resuspended by adding 35 ml of autoclaved demineralized water and then tubes were vortexed gently for 5 seconds to detach any cells sticking to tube walls. This process was repeated four times for each tube. After the fifth centrifugation, the cells were left in a volume of 5 ml (i.e. no water was added) but still vortexed gently. Hence, this washing process had the effect of diluting the nutrient solution originally present in the tube 4096-fold, effectively leaving the cells nutrient-deprived. The five tubes of concentrated cells per strain were then pooled into a single initial tube. A volume containing approximately 200 000 cells was placed in a 12 ml polystyrene culture tube and autoclaved demineralized water was added to increase the total volume to 2 ml. Three replicate tubes were set up for each strain. For each replicate, pictures were taken at 14 time steps up to 408 h (every four hours during the first 24 h, this delay increasing to 24 h and 48 h in the course of the experiment).

From the raw cell measurements at the replicate level, we estimated five major variables describing the changes in cell density and shape: survival as a density sum over time, mean and variance of maximal cell elongation, elongation persistence as a sum of cell elongation over time, and the frequency of putative disperser morphs after 8 h, time when the maximal elongation was observed (Table 2; see also Figure 6 and Additional file 1).

### Dispersal in presence of nutrients

Dispersal rate was measured using a two patch system setup consisting of two tubes (1.5 ml polypropylene test tubes, Greiner Bio-One) connected by a small horizontal pipe (silicone Tygon tubing, 6 mm diameter, 17 mm long) inserted through a hole drilled on the side of each tube. For each setup, a cell suspension volume corresponding to 300 000 cells of a given strain was taken from a culture tube (the initial tube) and introduced in one patch (the start tube), by the use of a pipette. By pipette we then filled up the target tube with cell-free nutrient medium, filling up in this way also the connecting tube and the remaining free space in the start tube. Both start and target tube contained 1 ml in the filled-up state. The setup was then incubated at 24°C. Pilot experiments using an ink solution instead of a cell suspension had verified that filling up the setup and placing it in the incubator did not induce perturbations that could displace cells from the start patch.

After 17 h we estimated the number of cells that had swum from the start to the target patch. This was done by closing both ends of the connecting tube with clamps and then pipetting off the solution in both start and target tubes and taking pictures for the two tubes. The experiment was performed over several days in a semi-block design due to constraints on manipulation time. A total of six replicates were done for each strain, in the manner of three replicates per strain for two out of four blocks (dates).

From the raw cell measurements, we estimated three major variables at the replicate level: initial cell shape, dispersal rate and cell elongation (Table 2).

### Single cell colonization capacity in presence of nutrients

To assess colonization capacity, individual cells were isolated from a one-week old cell culture tube using a handheld 10 μl pipetteman and a binocular dissection microscope. Each cell was placed separately in a 12 ml test tube with nutrient medium (the new patch to be colonized), and 10 replicates (10 different tubes) were created per strain. The entire set-up was replicated one week later, leading to a total of 20 single-cell tube experiments per strain. In the second experiment, however, some tubes became infected and were discarded. All tubes were incubated at standard temperatures in the incubator for eight days, after which the success of single cells to colonize the new patches by surviving and undergoing multiple cell divisions was determined.

To determine colonization success we took out a 10 μl aliquot from each experimental tube, after gentle vortexing, placed it on a slide and counted the number of cells directly under binoculars. We then assigned a 1 or a 0 to each experimental tube according to whether any cells were found or not (presence of at least one cell *versus *none), and calculated (over replicate tubes) the probability of successful colonization for each strain.

### Statistical analyses

The basic data for studying life-history trait covariation between strains were the estimates at the replicate level of 14 parameters (Table 2). Covariation between these parameters was studied through Spearman's correlation [97] and principal component analysis [98], and differences between strains by generalized linear models [99] and discriminant analyses [98], all implemented using SAS software.

Because replicates of a given strain were not linked between experiments, we used a permutation at the replicate level procedure to correlate parameters from different experiments. This procedure prevented discarding the information on variation between replicates of a given strain. The replicates of a given strain were randomly associated across experiments 1000 times, and a correlation was computed for each random association. The mean Spearman's correlation (r) was reported as covariation measure between the two parameters studied. Statistical significance of this correlation (showing that it differed significantly from zero), however, was based on the probability of obtaining the observed proportion of significant correlations by chance (in the 1000 simulations). The distribution of this proportion of significant correlations under the null hypothesis of no correlation was also obtained by resampling, with observed values randomized for replicate and strain, breaking any existing correlation. In total, 1000 such random associations were done and the proportion of significant correlations computed. This procedure was repeated another 1000 times, and the P-value for the test was computed as the proportion of random associations with a proportion of significant correlations greater than or equal to the one observed.

## Authors' contributions

The work presented here was carried out in collaboration between all authors. EJF, NS and JC defined the research theme. EJF and NS designed methods and experiments, carried out the laboratory experiments, analyzed the data, interpreted the results and wrote the paper. PM and AG co-designed the dispersal and colonization experiments, and co-worked on associated data collection and their interpretation. JC co-designed experiments, discussed analyses, interpretation, and presentation. All authors have contributed to, seen and approved the manuscript.

## Supplementary Material

Additional file 1 Fast, directional swimming behavior of dispersal morphs compared to other cells. This video clip shows the rapid, directional swimming behavior of *T. thermophila *dispersal morphs compared to other cells. Click here for file
